# Towards a Low-Cost Solution for Gait Analysis Using Millimeter Wave Sensor and Machine Learning

**DOI:** 10.3390/s22155470

**Published:** 2022-07-22

**Authors:** Mubarak A. Alanazi, Abdullah K. Alhazmi, Osama Alsattam, Kara Gnau, Meghan Brown, Shannon Thiel, Kurt Jackson, Vamsy P. Chodavarapu

**Affiliations:** 1 Department of Electrical and Computer Engineering, University of Dayton, 300 College Park, Dayton, OH 45469, USA; alhazmia3@udayton.edu (A.K.A.); alsattamo1@udayton.edu (O.A.); vchodavarapu1@udayton.edu (V.P.C.); 2 Department of Physical Therapy, University of Dayton, 300 College Park, Dayton, OH 45469, USA; gnauk1@udayton.edu (K.G.); galloglym1@udayton.edu (M.B.); thiels1@udayton.edu (S.T.); kjackson1@udayton.edu (K.J.)

**Keywords:** human gait recognition, gait analysis, mmWave radar, machine learning, 3D point-cloud, IWR6843ISK-ODS, neural network, Nvidia Jetson nano

## Abstract

Human Activity Recognition (HAR) that includes gait analysis may be useful for various rehabilitation and telemonitoring applications. Current gait analysis methods, such as wearables or cameras, have privacy and operational constraints, especially when used with older adults. Millimeter-Wave (MMW) radar is a promising solution for gait applications because of its low-cost, better privacy, and resilience to ambient light and climate conditions. This paper presents a novel human gait analysis method that combines the micro-Doppler spectrogram and skeletal pose estimation using MMW radar for HAR. In our approach, we used the Texas Instruments IWR6843ISK-ODS MMW radar to obtain the micro-Doppler spectrogram and point clouds for 19 human joints. We developed a multilayer Convolutional Neural Network (CNN) to recognize and classify five different gait patterns with an accuracy of 95.7 to 98.8% using MMW radar data. During training of the CNN algorithm, we used the extracted 3D coordinates of 25 joints using the Kinect V2 sensor and compared them with the point clouds data to improve the estimation. Finally, we performed a real-time simulation to observe the point cloud behavior for different activities and validated our system against the ground truth values. The proposed method demonstrates the ability to distinguish between different human activities to obtain clinically relevant gait information.

## 1. Introduction

The United Nations Population Fund (UNFPA) report states that the population of older adults (>60) will increase to 2 billion by 2050 [1]. This population is more prone to developing neurodegenerative diseases, becoming frail, and having falls [2]. Continuous monitoring of mobility and activity levels could provide valuable data to help mitigate the impacts of disease and disability [3]. However, it is difficult to monitor one or more individuals continuously and unobtrusively at home or in institutional settings. Fortunately, recent technological advancements have paved the way for easier remote monitoring [4,5]. In this context, remote health monitoring by leveraging Machine Learning (ML) and Artificial Intelligence (AI) methods is becoming more common [6]. Such technologies can provide the ability to monitor patients remotely, provide health reports to doctors, and detect potentially serious events, such as falls [7,8].

Important applications of remote health monitoring include continuous monitoring of gait and recognition of different walking patterns and activities and events, such as sitting, standing, laying, and falls [9]. Currently, gait analysis requires an evaluation by a healthcare professional in a clinical setting, which can be costly and time-consuming. Remote gait analysis could help to detect changes and deviations from normal walking, which could help health professionals to assess injury, illness severity, or recovery over extended periods of time [10]. Human Activity Recognition (HAR) is the method of identifying or classifying different human activities, such as sitting, walking, running, or falling. To date, various technologies have been used for HAR, such as wearable sensors, cameras, and radars [11,12,13,14]. In general, HAR involves monitoring activity over extended periods of time; however, sensors and wearables are impacted by their limited battery life [15]. Wearable sensors also require users to remember to use them regularly [16]. Video cameras can provide monitoring over extended periods of time, but their performance depends on a clear line-of-sight and can be impacted by lighting and climate conditions [17]. Further, video cameras have privacy concerns for the user, making them less optimal for HAR [18].

Millimeter-Wave (MMW) radars have shown potential for HAR with high resolution using their broad bandwidth of operating frequencies from 30 to 300 GHz [19]. MMW radar can overcome the battery limitations of wearables and the privacy concerns of cameras when used as a plug-in device attached to a wall power outlet and can operate in a variety of climate and lighting conditions [20]. MMW radar generates a 3D point cloud representation of joints that can be recognized as human locomotion using AI algorithms. Currently, most HAR devices and clinical measures of gait focus on spatiotemporal parameters such as gait speed, cadence, and distance [21,22]. MMW could provide additional information about the quality of gait that is otherwise difficult to measure in community or free-living environments. Based on the potential benefits of MMW, this paper aims to present its use for human gait analysis using ML algorithms. The novel contributions of this paper include:

- The use of MMW radar and ML to recognize and classify different abnormal gait patterns commonly associated with frailty and disability, including walking with a limp, walking with a stooped posture, and walking with an assistive device.
- The use of a low-cost MMW radar prototype with competitive object localization and detection accuracy that can be used in both community and clinical settings.
- The use of micro-Doppler signatures and skeleton pose estimation techniques to achieve a gait pattern classification accuracy of 95.7 to 98.8%.

The rest of the paper is organized as follows: Section 2 provides the background and literature review. Section 3 explains the proposed MMW system, and the experimental setup is presented in Section 4. Section 5 discusses the results and discussion. Finally, Section 6 concludes the work.

## 2. Background and Literature Review

Gait analysis examines the different postures of a human body between consecutive foot strikes of the same foot (gait cycle). Motion analysis laboratories use infrared motion capture cameras and force plates, which are the current gold standard for gait analysis; however, they are impractical for long-term monitoring in community or general clinical settings because of their cost and scale. While laboratory gait analysis can accurately measure various parameters, such as joint angles, muscle force, step/stride length, body posture, and ground reaction forces [23], it does not automatically recognize or classify gait into commonly known abnormal patterns.

To overcome some of the limitations of laboratory-based gait analysis, wearable sensors such as accelerometers, gyroscopes, and magnetometers are becoming more common for gait analysis. The advantage of wearables over other techniques is their ability to take readings in free-living environments [23]. Furthermore, wearables are small and inexpensive compared to other technologies. Zebin et al. [24] used data from five lower body inertial measurement units (IMUs) and the Convolutional Neural Network (CNN) to accurately classify activities such as walking, stairs, sitting, standing, and lying. However, wearable sensors cannot currently recognize and classify different abnormal walking patterns.

Surface electromyography (sEMG) is commonly used during gait analysis and gives important information regarding muscle activity [25]. Rescio et al. [26] showed that sEMG improves pre-fall detection accuracy compared with wearable IMUs. However, even slightly incorrect placement of sEMG electrodes drastically reduces the detection accuracy. These challenges make sEMG impractical for long-term community-based gait analysis.

Portable gait mats placed on a hard floor have been used for gait analysis. These mats are equipped with pressure sensors that measure ground reaction forces. The mats are low cost, portable, and noninvasive. However, they are typically only 3–5 m in length and not applicable to community environments.

Acoustic tracking using ultrasonic pulses has been used in some studies [27,28]. However, it does not provide optimal results in the presence of noise. This technique also requires a direct line of sight [29,30].

Vision-based systems use a motion camera to capture a subject’s gait and translate it to a 3D computerized object [31,32]. Therefore, such systems are widely used to examine gait by measuring joint angles, joint locations, and mobility in 2D and 3D. Privacy is a significant concern with vision-based systems [9].

Zhao et al. [33] presented RF-Pose for human pose estimation. It estimates the human pose by creating a 2D dynamic skeleton stick figure during walking activity. However, it is challenging to predict and distinguish hand movements and joints using a 2D stick figure, especially when a subject is walking with external assistance, for example, using a cane or walker.

MMW radar has emerged as a promising technology that can complement or replace other gait analysis and HAR systems over extended periods of time. MMW radar is low-cost and noninvasive in nature and can work under a variety of operational conditions [34]. Furthermore, it offers remote monitoring without direct line of sight. MMW radar generates point cloud data, reducing privacy concerns, and the data can be processed using AI and ML algorithms for HAR. Sengupta et al. [35] proposed a voxelization method using radar and Natural Language Processing for pose estimation. That study estimated 25 skeleton points using MMW radar. One concern is the increased computation cost due to the high dimensionality of the input data. An and Ogras [36] reduced the input data dimension by mapping 5D time-series point cloud data from MMW radar to a lower dimension. They then used the convolution neural network (CNN) to estimate human joints accurately. In addition, micro-Doppler signal components can help to reveal gait information. For example, considering a walking person, the Time-Frequency Representations (TFR) of the radar backscattering depict the micro-Doppler components due to swinging arms and legs [37]. A normal gait generates a periodic signal in its TFR. However, changes in gait, such as falling, generate a Doppler shift that can help with HAR.

The proposed method combines skeleton pose estimation and micro-Doppler signatures to recognize and accurately classify five different gait patterns. The following gait patterns were used for our study because they represent common abnormal patterns among older adults.

(1) Normal Gait—Walking normally with good posture and without an assistive device.
(2) Limping—Many gait deficits lead to asymmetry in movement; limping is a common gait disorder characterized by asymmetry in step/stride length and lateral trunk movement. A similar pattern can be seen in people with hemiparesis due to disorders such as stroke, Multiple Sclerosis (MS), and brain injury [38].
(3) Stooped posture—A stooped posture is common in frail persons who have difficulty overcoming the postural demands of gravity. A stooped posture is also common with various neurological disorders, such as dementia and Parkinson’s Disease [39].
(4) Using a walker—Many people with gait and balance disorders use walkers. The ability to detect whether someone is using a walker can help to determine whether they are complying with their prescribed use or have adopted the device independently or whether they were using it at the time of a fall [40]. We used a front-wheel rolling walker in this work, because these are very commonly used in home and institutional settings.
(5) Using a cane—Many people with gait and balance disorders use canes. The ability to detect whether someone is using a cane can help to determine whether they are complying with their prescribed use or have adopted the device independently [40].

## 3. Proposed MMW Radar Gait System

**Figure 1:**
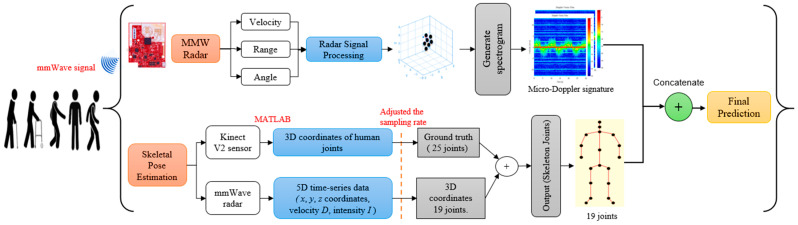
A flow diagram of the MMW radar gait system.

The proposed MMW radar gait system is a low-cost solution for distinguishing different human activities and gait patterns. It consists of several steps. First, the system generates the micro-Doppler signatures based on point cloud data from the MMW radar. Then, a CNN algorithm identifies different human activities using micro-Doppler as an input. To train the CNN algorithm to accurately estimate the locations of human joints, we used 3D joint coordinates obtained from the Microsoft Kinect V2 sensor as ground truth. Then, the trained model reconstructed 19 human joints and their skeleton from the point cloud generated by the MMW radar. Figure 1 illustrates the flow diagram of the MMW radar gait system.

### 3.1. Detection Using Micro-Doppler Signatures

**Figure 2:**
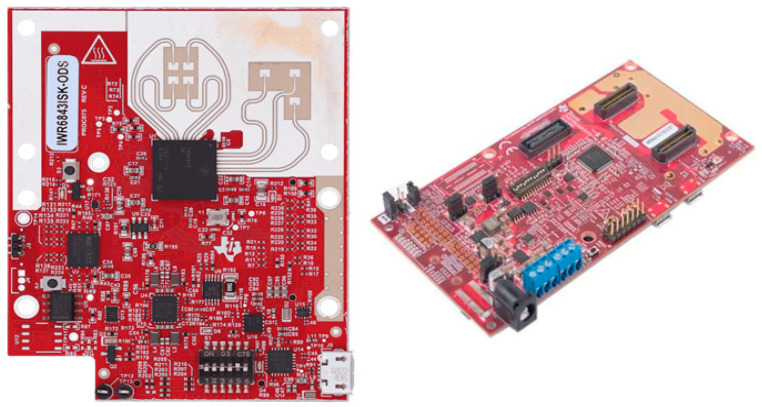
TI IWR6843ISK-ODS MMW radar (**left**), and MMWAVEICBOOST evaluation board (**right**).

The MMW signals from the radar generate various scattering points after reflecting from a human body. The scattering points of different body parts create the point cloud specific to that part of the body. Generally, these points have different velocities or Doppler shifts because of movements, such as the movement of legs or arms. For example, the right leg will have velocity in the opposite direction to the left leg during a walk or run. Therefore, the Doppler of every point cloud will be different for different body parts. Hence, each activity creates a unique micro-Doppler signature calculated using the velocity of scattered points over time. For this study, we used the Texas Instruments (TI) IWR6843ISK-ODS MMW radar, as shown in Figure 2 [41].

#### TI IWR6843ISK-ODS MMW Radar

The TI IWR6843ISK-ODS MMW radar uses Frequency-Modulated Continuous-Wave (FMCW) to precisely measure the velocity range and angle by sending continuous frequency-modulated signals. It consists of three transmitting and four receiving antennas with a 120° field of view with a range of approximately 12 m, enough to cover areas of various residential and clinical settings.

**Figure 3:**
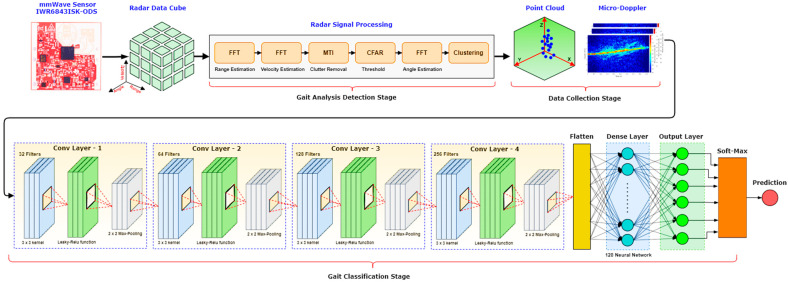
An overview of the proposed HAR detection method Using Micro-Doppler signatures.

The micro-Doppler Signatures steps are illustrated in Figure 3. At the start, the IWR6843ISK-ODS MMW radar sends multiple FMCW chirps with a 60.75 GHz carrier frequency. The chirps help to calculate different parameters, such as velocity, range, and angle. For example, velocity is calculated by finding the Doppler shift across Coherent Processing intervals (CPIs). In contrast, beamforming using multiple antennas helps to analyze the angle. In the second step, the measured velocity, range, and angle are transformed into a 3D data cube, as illustrated in Figure 3. Radar processing consists of five steps:

1. Range and velocity estimation is performed in the first step using the Fast Fourier Transform (FFT).
2. Moving Target Indication (MTI) removes the static clutter points (surrounding reflections) from the data.
3. The Constant False Alarm Rate (CFAR) detects static points among the noisy data in the fourth step.
4. The fifth step includes angle estimation using the FFT.
5. Finally, Density-Based Spatial Clustering of Applications with Noise (DBSCAN) is applied to separate the scattered points into different categories, such as regular walking or walking with a limp [42].

The Kalman filter tracks subjects’ movements and associates relevant scattered points with different activities, thus making point clouds for each activity.

**Figure 4:**
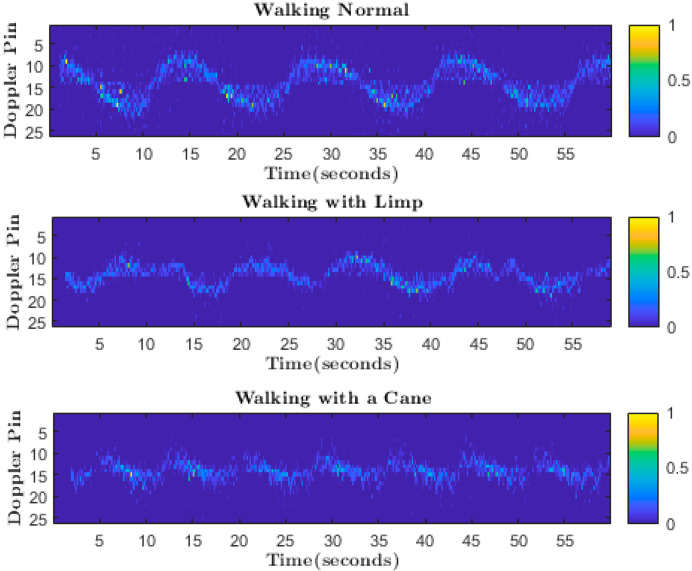
Micro-Doppler signature of different activities.

We used a sliding window to collect Doppler features over time. In the proposed method, the attenuation of the scatter point intensity is compensated for due to the range effect and normalized to one. After that, these points are fed to the CNN for training. The normalized point cloud’s Doppler pattern acts as a signature for different activities and helps to classify different activities. The micro-Doppler signature of each activity differs, as shown in Figure 4.

The last step of this approach is to distinguish five different activities based on the micro-Doppler signature of each activity. For that, we use a four-layered CNN, as illustrated in Figure 3, due to its high accuracy and low loss rate. We experimented with multiple CNN layers during our analysis, and our results show that optimal results are achieved with four convolution layers. Figure 3 shows that each layer consists of a 3 × 3 kernel, with depths of 32, 64, 128, and 256, respectively. Further, we use the Leaky-Rectified Linear Activation Unit (*ReLU*) to mitigate the dying ReLU impact that sends a few neurons to the inactive state [43]. Next to *ReLU*, a 2D max-pooling layer reduces the computation complexity by downsampling the previous layer’s output while keeping the most dominant features. This is achieved by sliding a 2 × 2 window across the CNN output and returning the maximum pixel value in its stride. We also use dropout regularization between layers with a dropout probability of 5% [35]. This reduces the number of parameters at each epoch, resulting in lower computation and a high training speed. The output of the CNN layers is flattened to a 1D vector, as depicted in the Figure 3. After passing through the dense layer, the final output will have k nodes against k features/activities we are planning to classify. Finally, these outputs are normalized and associated with each class/activity probability using the softmax function. Hence, the class with maximum probability is the predicted human activity. In our study, we combined these micro-Doppler signatures (using CNN) results with the joint estimation techniques to obtain precise results.

### 3.2. Skeletal Pose Estimation Technique

**Figure 5:**
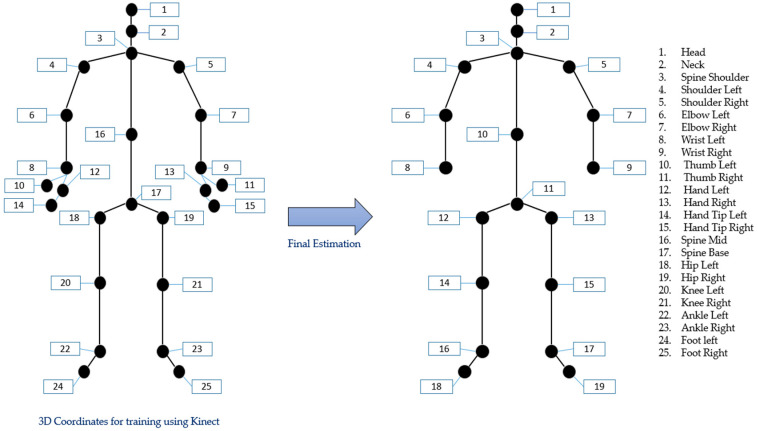
The training and estimated joint positions.

**Figure 6:**
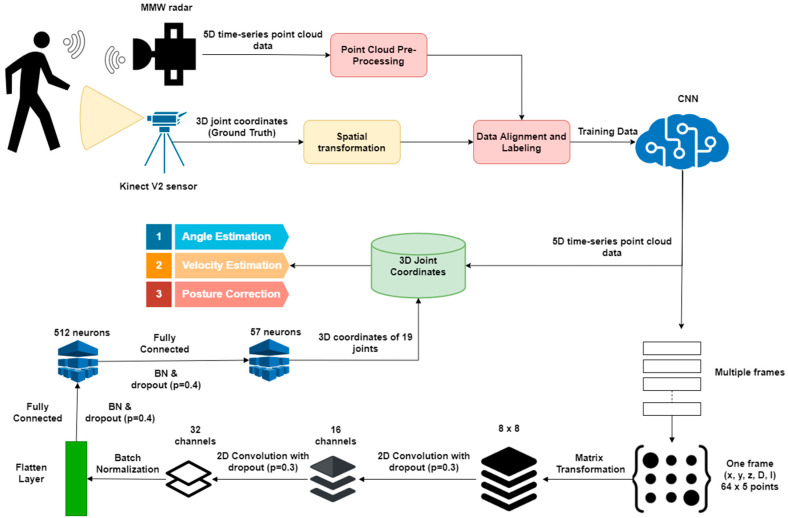
Joint estimation using the 5D time-series point cloud.

The skeletal pose estimation technique combines the 5D time-series point cloud data from MMW radar with the 3D joint coordinate data from the Kinect V2 sensor. Kinect provides 25 joint coordinates for training, while CNN combines this training data with time-series data to accurately predict the locations of 19 human joints, as shown in Figure 5. The flow diagram of the used method is illustrated in Figure 6.

TI’s MMW extracts the 5D time-series data to frames, including the (x, y, z) coordinates, reflection intensity, and Doppler shift, using reflected chirp signals. However, the reflected chirp signals arrive at the radar randomly due to unexpected body movements or delays. As a result, the frames do not have consistent point locations. To achieve better prediction accuracy using the CNN, the frames should have consistent data locations and shapes. Therefore, we performed data preprocessing using matrix transformation and sorting algorithms. The transformation also reshapes the data in the matrix, thus, making it an ideal input for the CNN. Furthermore, it caters for the outliers caused by scattering in the training process, because such phenomena are inevitable in real applications.

The second step involves converting these frames to 19 joint positions using the CNN, as shown in Figure 5. The proposed CNN algorithm uses a 5-channel feature map as the input. It consists of two convolution layers, one flattening layer, and two fully connected layers. Next, multiple Batch Normalization (BN) layers are used after the convolution layer and between the fully connected layers to avoid major data distribution changes.

The input data obtained by the transformation matrix are passed through convolution layers with 16 and 32 channels, respectively. Each layer is followed by a dropout layer (with probabilities of 0.3 and 0.4) to avoid high dependency on a particular neuron. The output of these layers is passed through a flattering layer, which creates an input vector for fully connected layers. The first fully connected layer consists of 512 neurons, while the last layer consists of 57 neurons of 19 joints (3D points for each point). The fully connected layers also use dropout layers to eliminate dependency on a particular neuron.

The training of the proposed CNN requires the ground-truth value. Therefore, the Kinect V2 sensor was used to measure the referencing coordinates. Generally, it is difficult to predict hand motions of people using canes or walkers. Therefore, we extracted six extra ground truth points using the Kinect system, as shown in Figure 6. For example, we estimated locations 9, 11, 13, and 15 for the right hand. The point with the highest prediction was considered the right wrist during the final estimation, so all other points were discarded.

**Figure 7:**
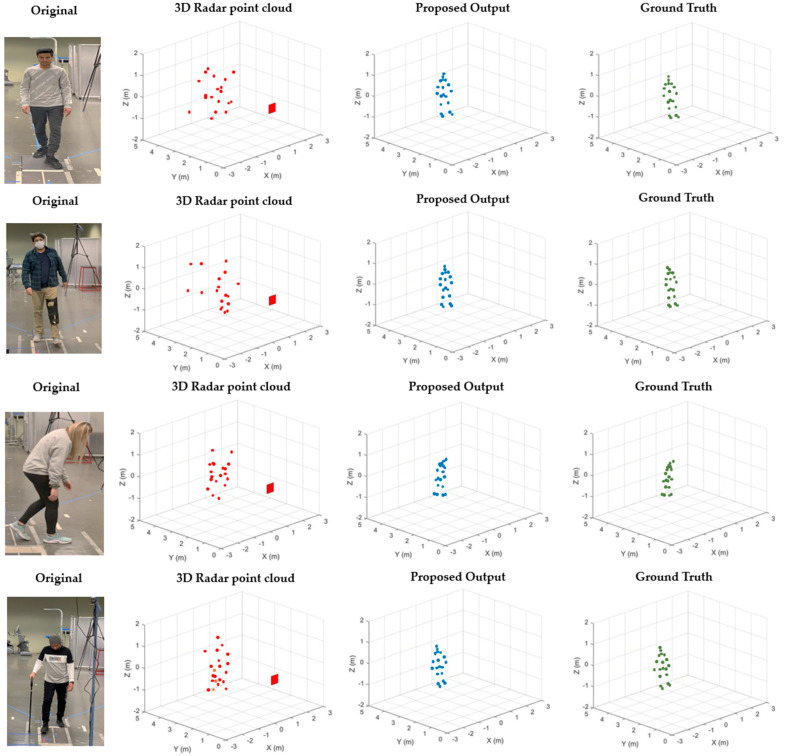

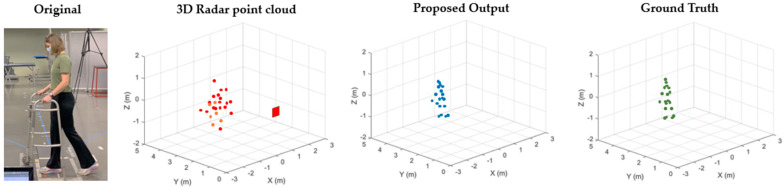
Proposed system for reconstructing human joints from a point cloud. (**Left**) (MMW radar point clouds), (**middle**) (Proposed estimation), (**right**) (Kinect ground truth).

We placed the MMW radar and Kinect V2 sensor during the experiment to capture the data to train our model. That configuration led to a spatial offset in the *x*-axis that was addressed during the preprocessing phase. We ensured the frame alignment by connecting both devices to the same laptop and timestamping the data frames from each device. Our proposed method tracks the (x, y, z) coordinates using the referencing/ground truth coordinates of Kinect to compare our model outputs during the real simulation, as shown in Figure 7.

Figure 7 illustrates the point clouds, ground truth values, and estimated values obtained using the proposed method. Figure 7 shows that the MMW radar extracted point clouds have sparsity, due to the limitations of radio wavelength and inherent noise, which makes it difficult to estimate the activity or pose accurately. The proposed MMW gait system accurately estimates various joint locations for different activities and is closer to ground truth values, as visualized in Figure 7.

Later, these estimations are combined with the micro-Doppler estimate to predict five different human activities, as discussed in Algorithm 1.

### 3.3. MMW-Based HAR Algorithms

This algorithm identifies different human activities using micro-Doppler as an input for MMW [${ℳ}_{i}\;:i=1,2,3,4,5$] with five different walking postures. First, it generates the radar signals of inputs in the form of d= Range, v= Velocity, and θ= Angle. The range is used to locate the users, and the distance from the radar can be calculated by d:

(1) $$d=\frac{f_{1F}\times c\times T_{c}}{2\times B}=\frac{f_{1F}\times c}{2\times S}$$

where $f_{1F}$ represents the IF frequency (time-domain signals), c  is the speed of light, the duration of each chirp signal is denoted by $T_{c}$, and B represents the reflected signals.

To distinguish between multiple users, the velocity of each user is collected and denoted by ν:

(2) $$\nu =\frac{\omega \times \lambda }{4\pi \times T_{c}}$$

where ω represent the phase difference, and λ represents the wavelength.

To find the exact position in the spatial Cartesian coordinates system, we derive the Angle of Arrival Estimation (AoA) =θ using multiple neighboring receiving antennas $d_{i}-R_{x}$. This AoA=θ is derived using the relation

(3) $$\theta =\sin^{-1}\left(\frac{\omega \times \lambda }{2\pi \times d_{i}-R_{x}}\right)$$

**Algorithm 1:** The MMW-based HAR algorithm using the CNN.**Require:** MMW $\left[M_{i}:i=1,\;2,\;3,\;4,\;5\right]$
**Ensure:** Walking Posture $W_{p}\left[W_{p}:p=1,\;2,\;3,\;4,\;5\right]$
    **for**
$M_{i}\leftarrow 1:5$ **do**
          read radar signal           **estimate** d←Range
          **estimate** v←Velocity
          **estimate** θ←Angle    **end for**
    **for** $M_{i}\leftarrow 1:5$ **do** read MMW and Kinect V2 data
          **extract** x, y, z coordinates
          **extract 5D points**
j← 19 points
          **extract 3D points**
k← 25 points
          **estimate** skeleton joints $\left[S_{j}=j-6\right]$            ⊳ total 19 points
          **estimate** $S_{j}+MD_{p}S$                                  $⊳MD_{p}S:$ micro-Doppler Signatures
          **get** prediction probabilities $W_{p}\left[W_{p}:p=1,\;2,\;3,\;4,\;5\right]$
  **end for**
  **Read**
$W_{p}\left[W_{p}:p=1,\;2,\;3,\;4,\;5\right]$
  **Predict** Walking Posture 

After that, a four-layered CNN identifies different human activities using micro-Doppler Signatures $MD_{p}S$ as the input. The data alignment and labelling are calculated using ν and time (t) as

(4) $$MD_{p}S=\frac{\nu }{t}$$

The prediction of skeletal pose estimation is further based on the Kinect V2 sensor and MMW radar values. The Kinect V2 sensor records 25 human joints=j in a 3D coordinate system, whereas the MMW radar records 19 human joints with five coordinates x, y, z, D, I. Then, these joints are subtracted from the joints of the 3D system using the formula $S_{j}=j-6$. These six subtracted values are actually the joints of the hand recorded twice.

Finally, the walking prediction $W_{p}\left[W_{p}:p=1,\;2,\;3,\;4,\;5\right]$ is done using the relation

(5) $$W_{p}=S_{j}+MD_{p}S$$

DBSCAN is applied to separate the scattered points into different categories, such as normal walking or walking with a limb.

- W1: walking normal.
- W2: walking with stooped posture.
- W3: walking with limp.
- W4: walking with a walker.
- W5: walking with a cane.

## 4. Experiment Setup

Table 1 lists the configuration parameters of the TI’s IWR6843ISK-ODS MMW radar used to classify human activities into the five working classes (W1, W2, W3, W4, W5) listed above.

We proposed a clinical study that was approved by the Institutional Review Board (IRB) at the University of Dayton and recruited approximately 74 participants. The participants’ demographics are provided in Table 2. The obtained datasets were used to train our deep learning CNN model and apply it in real-time.

**Figure 8:**
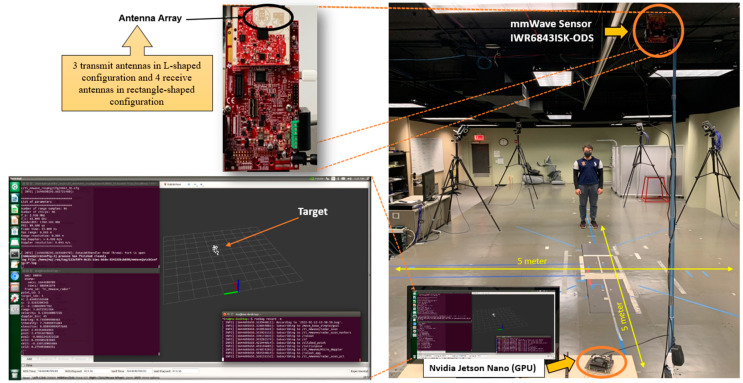
Experiment Setup.

The testing environment is shown in Figure 8. The environment consisted of a MMW radar sensor placed on top of a tripod with a height of 2 m and rotated with a tilt angle of 15 degrees for better area coverage. Based on the Robotic Operating System (ROS) on the Ubuntu-running Nvidia Jetson nano platform, we developed an interface program to connect the TI MMWAVEICBOOST and collect the radar 3D point cloud over the USB port.

### Data Collection and Labelling

**Figure 9:**
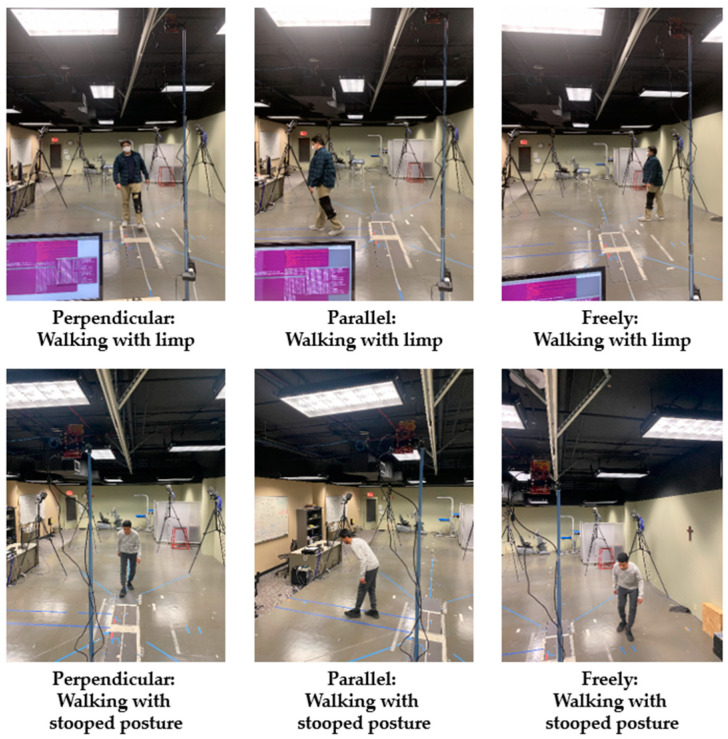
Data collection in the laboratory environment.

The data were recorded in three scenarios, as shown in Figure 9. We asked participants to walk perpendicularly in front of the sensor for one minute. This step included walking forward and backwards. Then, we asked participants to walk parallel to the sensor for one minute, walking forward and backwards. Finally, we asked the participants to walk freely in front of the sensor for one minute. The aim was to collect more data from different angles to improve the model’s training at different angles.

**Figure 10:**
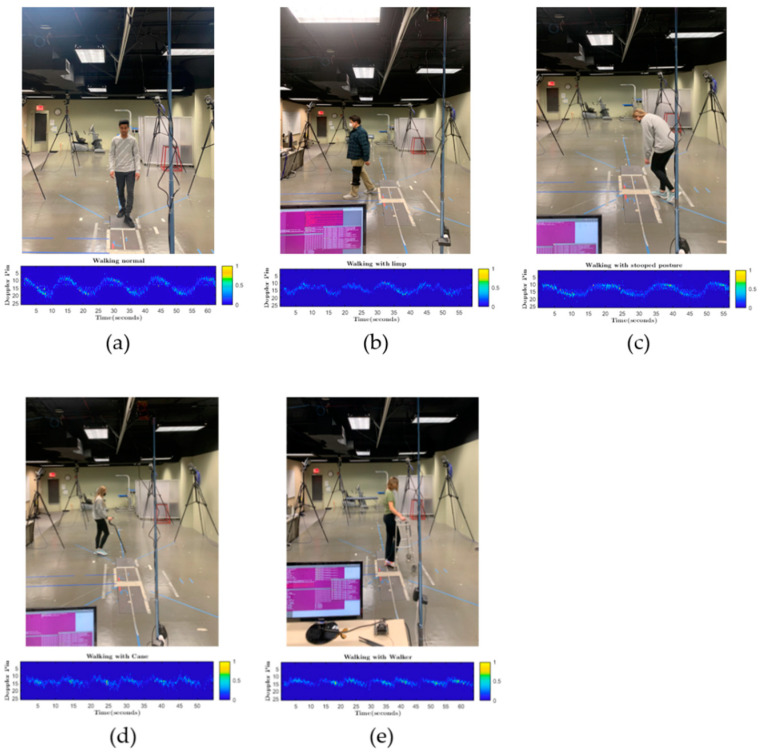
Micro-Doppler spectrogram produced during the data collection process for different activities. (**a**) walking normal, (**b**) walking with limp, (**c**) walking with stooped posture, (**d**) walking with a cane, (**e**) walking with a walker.

Figure 9 only shows data collection for limping and stooped posture due to space limitations. We used a python script to convert the files into CSV files containing all measurement values, such as time, target_idx, x, y, z, range, velocity, doppler_bin, bearing, intensity, elevation, posX, posY, posZ, velX, velY, and velZ. The micro-Doppler data collection process is illustrated in Figure 10.

**Figure 11:**
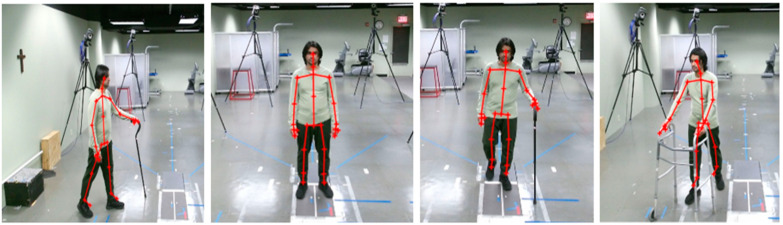
Joint estimation during the data collection process for different activities.

Similarly, data collection using the Kinect V2 sensor to train the model for reconstruction of the human joints is presented in Figure 11.

## 5. Results & Discussion

First, we performed a real-time simulation to observe the point cloud behavior for different activities and then validated our system against the ground-truth values. Second, we calculated the training and prediction accuracy levels.

### 5.1. Monitoring Individual Activity

**Figure 12:**
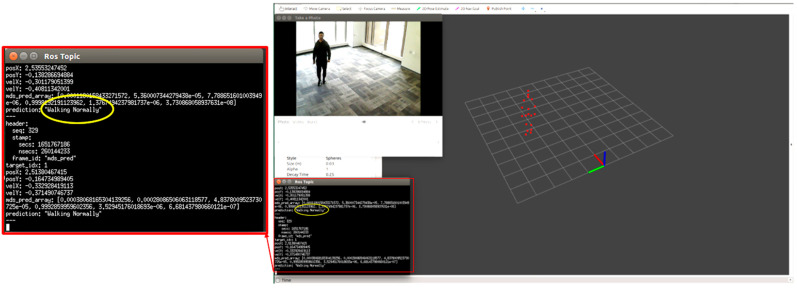
Subject walking normally.

**Figure 13:**
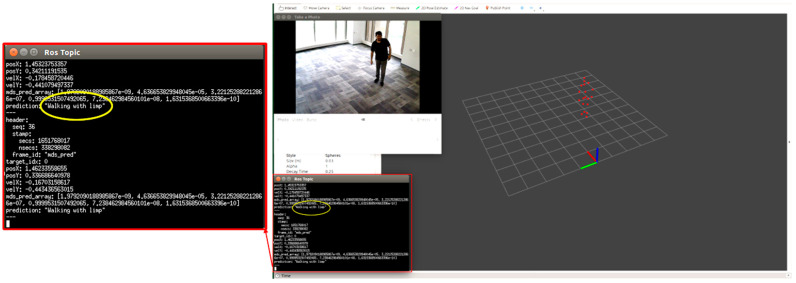
Subject limping.

**Figure 14:**
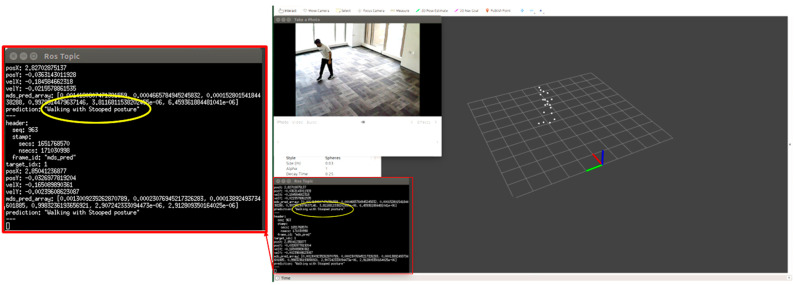
Subject walking with a stooped posture.

**Figure 15:**
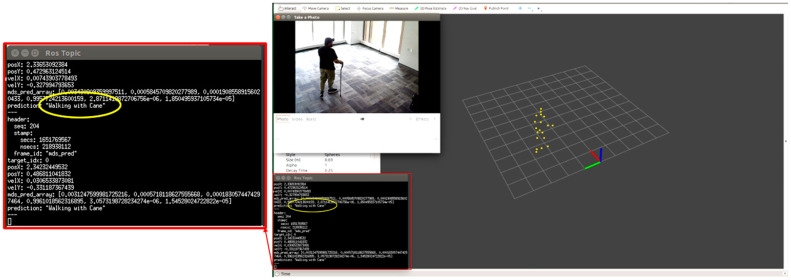
Subject walking with a cane.

**Figure 16:**
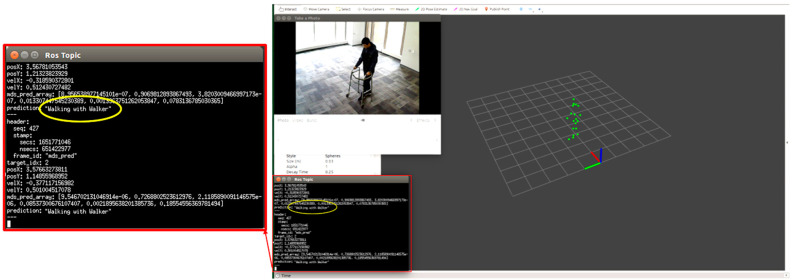
Subject walking with a walker.

We tested each participant condition/activity separately, for example, normal walking, limping, and stooped posture. We monitored the point cloud and the prediction message on the MMW system dashboard as circled by yellow color. For example, Figure 12 shows a person walking normally in the library environment. We can visualize that the generated point cloud structure is similar to the ground truth value or actual standard walking pose. Similarly, the MMW radar gait dashboard classifies it as “walking normally”, as highlighted by the yellow circle. The same trend is visible for the remaining activities illustrated in Figure 13, Figure 14, Figure 15 and Figure 16.

### 5.2. Monitoring Multiple Activities

**Figure 17:**
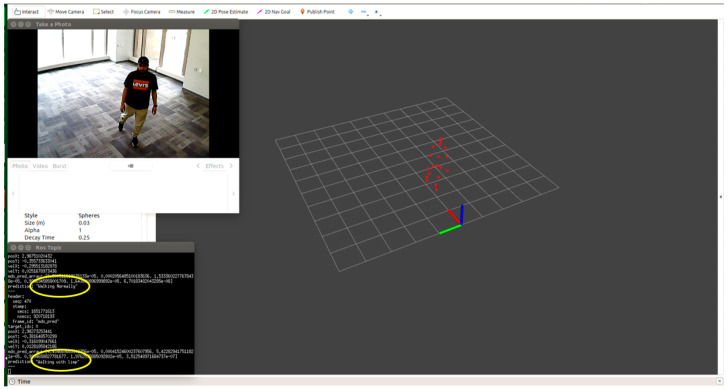
Walking normally and then limping.

**Figure 18:**
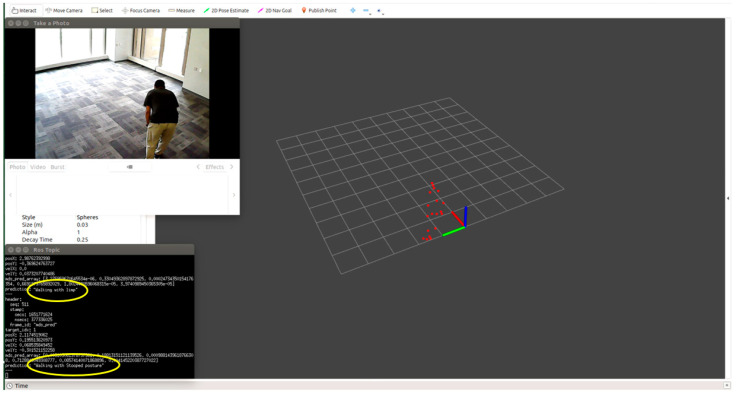
Limping and then stooped posture.

In this step, we examined the detection of multiple activities for an individual. The participant was asked to perform multiple activities, such as walking normally and then limping to monitor the sequences of change in the output and the changes in the prediction message. As an example, we show the following scenarios:

- Scenario 1: Walking normally and then limping (Figure 17).
- Scenario 2: Limping and then stooped posture (Figure 18).

### 5.3. Monitoring Different Subjects with Different Activities

**Figure 19:**
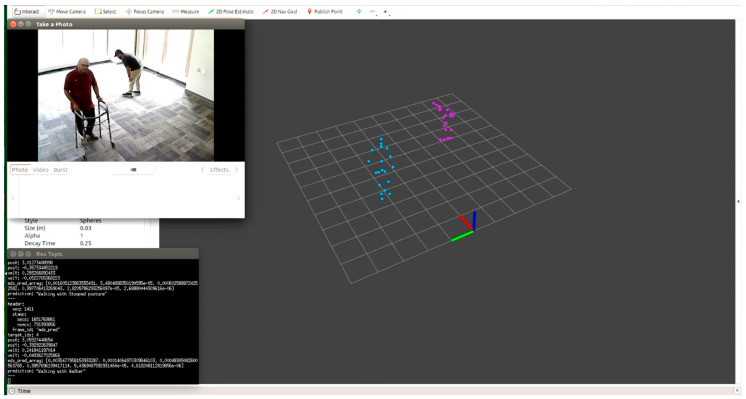
Two subjects, one walking with a walker and one walking with a stooped posture.

**Figure 20:**
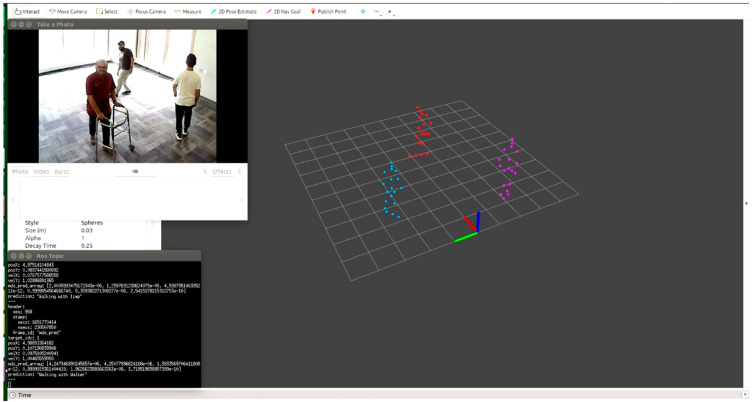
Three subjects, one walking with a walker, one walking normally, and one limping.

The proposed system has the ability to detect multiple subjects with different positions/activities at the same time. Therefore, as examples, we examined the results by considering the following scenarios:

- Scenario 1: Two subjects, one walking with a walker and one walking with a stooped posture (Figure 19).
- Scenario 2: Three subjects, one walking with a walker, one walking normally, and one limping (Figure 20).

#### 5.3.1. The Accuracy Evaluation and Time Analysis

Table 3 compares the accuracy of the predicted 3D joint coordinates against the ground truth. Here, we computed the Mean Absolute Error (MAE) and Root-Mean-Squared Error (RMSE) for x, y and z coordinates of different joints, as illustrated in Table 3. We trained five different models and averaged them to eliminate the system errors. The average MAE for all 19 joints was 5.86, 2.98, and 5.49 cm for the x, y, and z axes, respectively. Similarly, the average RMSE was 8.66, 4.45, and 7.75 cm for those coordinate axes, respectively.

The results suggest that the x and z axes have slightly larger errors than the *y*-axis since the observed movements involved more horizontal and vertical displacement of all body parts. In contrast, the error along the *y*-axis was minimal (2.13–4.13 cm) due to the smaller displacement in depth. Generally, most joints’ MAE errors were smaller than 8 cm. The most notable exceptions were the right and left wrist joints. As an explanation, the joints related to the hands need a higher resolution for localization. Since the MMW radar’s range resolution is 0.084 m at 1780.393 MHz, as mentioned in Table 1, it is difficult for the model to reconstruct these points precisely.

We used the Nvidia Jetson nano, which includes a Graphical Processing Unit (128-core Maxwell) and a Central Processing Unit (Quad-core ARM A57 @ 1.43 GHz) which offers the required computational power for HAR [44]. For different configurations, the total inference time required to process all 50,400 frames ranged from 2.2 s to 3.7s. The total power consumption ranged from 3900.2 mW to 5763.4 mW. The average frame processing time ranged from 54.3 μs to 92.5 μs.

#### 5.3.2. Prediction Accuracy

**Figure 21:**
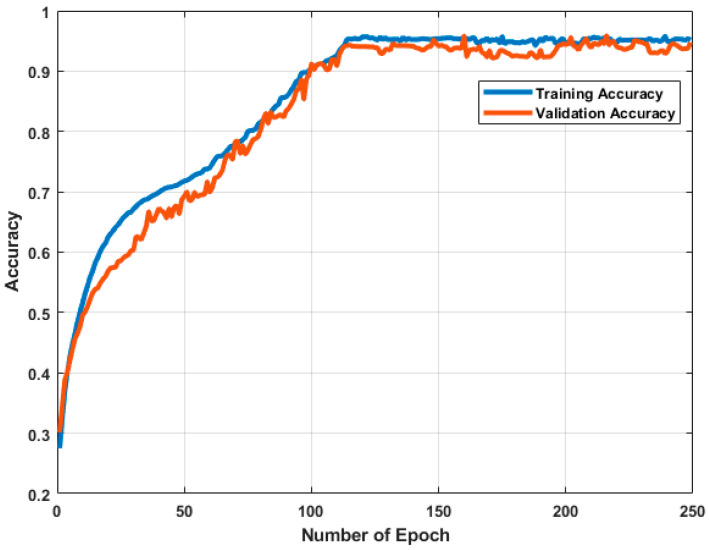
The results for the training and validation data (Accuracy).

**Figure 22:**
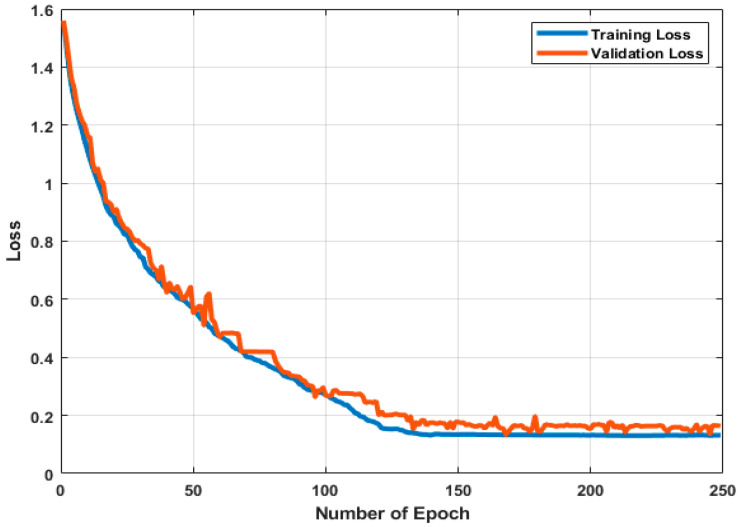
The results for the training and validation data (Loss Function).

Figure 21 illustrates the training and validation accuracy during the training process. In contrast, Figure 22 visualizes the training and validation losses. Figure 21 and Figure 22 show that the model adjusted the weights to identify all the activities correctly. Furthermore, the accuracy and loss function improved with more epochs, indicating that the model can generalize and accurately predict the outcome on validation data.

**Figure 23:**
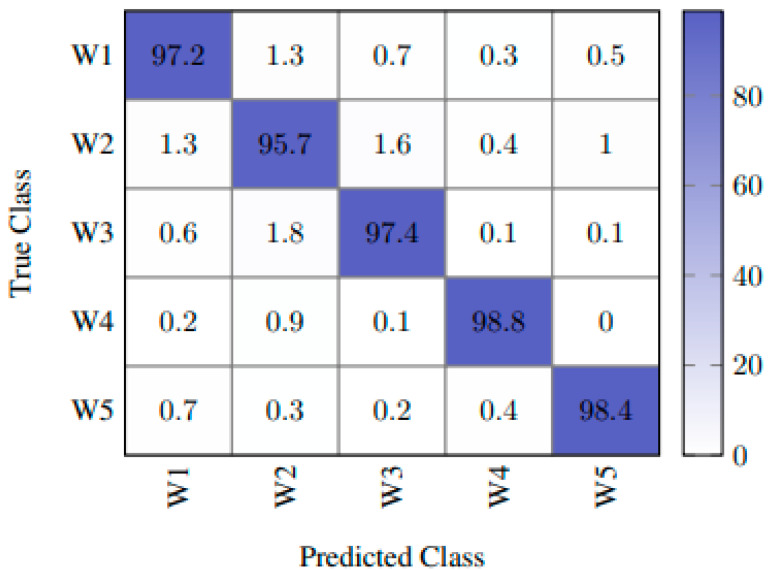
Confusion matrix for five different human activities.

Lastly, Figure 23 plots the confusion matrix for all activities. The first element shows that our proposed method predicted normal walking (W1) with an accuracy of 97.2%. In contrast, it predicted normal walking as a stooped posture 1.3% of the time. Similarly, it confused normal walking, limping, and cane and walker walking 0.6%, 0.2%, and 0.7% time, respectively.

The proposed method attained the highest prediction accuracy of 98.8% for walking with a walker, closely followed by a prediction accuracy of 98.4% for walking with a cane. However, the first three activities (W1, W2, and W3) demonstrated slightly lower prediction accuracy levels, because their gait patterns overlap sometimes. Nevertheless, the lowest accuracy of 95.7% is still clinically relevant for HAR for rehabilitation or remote monitoring purposes.

## 6. Conclusions

This study demonstrated the ability of the MMW radar system to accurately identify five different gait patterns. The proposed system combines pose estimation techniques with micro-Doppler signatures obtained by the low-cost radar system. The use of MMW radar for gait analysis preserves users’ privacy, does not require line of sight, can track multiple people at the same time, and can operate in varied environmental conditions. The information generated by this approach could be used to recognize other gait patterns and kinematic variables, such as joint angles.

This work has the potential to provide a number of practical clinical benefits, including the ability to track changes in the gait of one or more individuals over extended periods of time in both home and institutional settings. This would allow healthcare professionals to remotely monitor and assess the effectiveness of rehabilitation interventions outside the clinical setting and provide data that may indicate the need for additional interventions. In institutional settings, such as skilled nursing facilities, this system could provide data on the walking ability of residents to mitigate fall risk and to direct resources more effectively.

## Figures and Tables

**Table 1:** IWR6843ISK-ODS Radar Parameters.

Parameter	Physical Description
Start Frequency	60.75 GHz
Number of TX	3 TX
Number of RX	4 RX
Number of samples per chirp	96
Number of chirps	288
Maximum velocity	16.2 km/h
Velocity resolution	0.324 km/h
Idle time	30 µs
ADC valid start time	25 µs
F_s (Sampling frequency)	2.950 Msps
F_c (Central frequency)	63.008 GHz
Valid sweep Bandwidth (BW)	1780.393 MHz
$\mathrm{Periodicity}\;(T_{\mathrm{Frame}}$)	55 ms
$\mathrm{Max}\;\mathrm{unambiguous}\;\mathrm{range}\;(R_{\max}$)	8.083 m
Range resolution(∆R)	0.084 m
$\mathrm{Max}\;\mathrm{unambiguous}\;\mathrm{Doppler}\;(D_{\max}$)	±4.450 m/s
Doppler resolution (∆D)	0.093 m/s
Range Detection Threshold	15 dB
Doppler Detection Threshold	15 dB

**Table 2:** The participants’ distribution.

Parameter	Mean ± SD (Range)
Age	24 ± 7.36 (21–53)
Height (cm)	170 ± 5.55 (160–185.42)
Weight (kg)	75 ± 12.59 (55–115)
BMI	25.47 ± 4.36 (19.26–40.75)
Gender (M/F)	42/32

**Table 3:** Average Localization Error for 3D joint coordinates.

No. Point	Description	X (Horizontal) (cm)		Y (Depth) (cm)		Z (Vertical) (cm)	
		MAE	RMSE	MAE	RMSE	MAE	RMSE
1	Head	6.35	9.46	2.59	3.59	7.10	9.58
2	Neck	5.68	8.71	2.47	3.23	6.47	8.87
3	Spine Shoulder	5.51	8.46	2.13	3.02	6.30	8.61
4	Shoulder Left	5.82	8.80	2.28	3.28	5.77	8.01
5	Shoulder Right	5.64	8.58	2.66	4.02	6.01	8.11
6	Elbow Left	6.41	9.12	3.26	5.01	7.08	9.61
7	Elbow Right	6.85	9.63	3.61	5.62	7.30	9.81
8	Wrist Left	9.23	12.66	4.02	5.71	12.45	16.23
9	Wrist Right	9.62	13.14	4.13	6.14	13.03	16.52
10	Spine Mid	5.05	7.81	2.02	2.86	5.71	7.85
11	Spine Base	4.55	7.12	2.45	3.87	4.87	6.72
12	Hip Left	4.54	7.02	2.45	3.87	4.72	6.56
13	Hip Right	4.45	7.02	2.56	4.04	4.82	6.67
14	Knee Left	4.46	7.01	3.07	4.52	2.14	3.43
15	Knee Right	5.10	7.42	3.27	4.72	2.50	4.21
16	Ankle Left	4.45	7.08	3.08	4.54	2.23	3.42
17	Ankle Right	5.81	8.32	3.34	5.09	1.65	4.26
18	Foot Left	5.48	8.25	3.72	6.02	2.05	4.12
19	Foot Right	6.27	8.87	3.46	5.42	2.10	4.69
**19 points Average**		5.86	8.66	2.98	4.45	5.49	7.75

## Data Availability

The data presented in this study are available upon request from the corresponding author.
